# C-arm computed tomography parenchymal blood volume measurement in evaluation of hepatocellular carcinoma before transarterial chemoembolization with drug eluting beads

**DOI:** 10.1186/s40644-015-0057-x

**Published:** 2015-12-29

**Authors:** Roland Syha, Gerd Grözinger, Ulrich Grosse, Michael Maurer, Lars Zender, Marius Horger, Konstantin Nikolaou, Dominik Ketelsen

**Affiliations:** Department of Radiology, Diagnostic and Interventional Radiology, Eberhard-Karls-University, Hoppe-Seyler-Str. 3, 72076 Tübingen, Germany; Division of Translational Gastrointestinal Oncology, Department of Internal Medicine I, University of Tuebingen, Tuebingen, Germany

**Keywords:** Hepatocellular carcinoma, Transarterial chemoembolization, Drug eluting beads, C-arm computed tomography, Parenchymal blood volume

## Abstract

**Background:**

C-arm computed tomography (CT) guided intervention is an increasingly applied technique in transarterial chemoembolization (TACE) of hepatocellular carcinoma (HCC). The aim of this study was to analyse the value of parenchymal blood volume (PBV) maps acquired during C-arm CT acquisition, for pre-treatment evaluation and planning of TACE in HCC patients.

**Methods:**

A total of 64 HCC lesions in 29 patients (median age, 73 years, range, 62–77 years) were included in this retrospective study. All patients received cross-sectional imaging (MRI or CT) prior to TACE and C-arm CT PBV measurement acquisition before performing TACE. Results of cross-sectional imaging regarding the number of HCC lesions and maximum diameter were compared to PBV–maps. Number of lesions and tumour feeding vessels detected in PBV-maps were compared to conventional angiography. Results of PBV were analysed concerning different tumour morphologies (pre-treated, encapsulated and diffuse).

**Results:**

Pre-interventional cross-sectional imaging and PBV maps showed an excellent agreement in lesion diameter (*p* = 0.88, MD = −0.28 mm) and number of detected lesions (κ = 1.0). Compared to conventional angiography, PBV maps showed an increased number of detected lesions (κ = 0.77, *p* = 0.001) and tumour feeding vessels (κ = 0.71, *p* < 0.0001). Diffuse HCC lesion revealed a significantly lower PBV compared to encapsulated lesions (*p* = 0.0001).

**Conclusions:**

C-arm CT acquired PBV measurements detect HCC tumours with a lesion detectability comparable to pre-interventional cross-sectional imaging. Furthermore, this technique facilitates TACE, allowing a more precise localization of HCC lesions and tumour feeding vessels compared to conventional angiography. Additionally, calculated PBV values enable a real time quantitative assessment of tumour perfusion.

## Background

Hepatocellular carcinoma (HCC) is one of the leading tumour entities in industrialized countries. Within the standard of care, transarterial chemoembolization (TACE) is recommended as the first-line treatment for compensated patients with intermediate-stage [BCLC (Barcelona classification of liver cancer) stage B]. Furthermore TACE might be suitable in early stage HCC as bridging to transplantation or adjuvant therapy (BCLC A) [1].

Detection of smaller or less vascularised liver tumours is reported to be limited using conventional angiography, especially in liver parenchyma with inhomogeneous perfusion such as cirrhotic liver tissue [2–4]. Besides conventional angiography peri-procedural C-arm computed tomography (CT) is increasingly used during TACE. The aim of C-arm CT with a flat detector panel as part of an angiographic suite is to improve the performance of intraarterial treatments such as TACE or selective internal radiation therapy [5]. It allows intraprocedural acquisition of a volume of interest and post-processing including maximum intensity projections and multiplanar reconstructions comparable to conventional CT. C-arm CT enables acquisition of unenhanced and contrast enhanced images of liver parenchyma during different parenchymal phases [5]. Especially regarding TACE, C-arm CT increases detectability of HCC lesions and tumour feeding vessels [6]. Additional information is gained in over 80 % of patients undergoing TACE [7], and a substantial change of treatment procedure is described in up to 30 % of patients [8]. The introduction of dual phase C-arm CT increases the amount of information acquired as different contrast phases facilitate lesion detectability [9]. Acquisition of unenhanced and contrast enhanced C-arm CT furthermore enables acquisition of parenchymal blood volume (PBV) information and allows assessment of three dimensional (3D) cross-sectional images including native and contrast-enhanced anatomical as well as perfusion based data after arterial or venous injection of contrast medium [10]. Its application in neurological imaging has been reported [11] to show benefits as a monitoring tool during the procedure but also as an important tool during the pre-selection process of patients.

This is of great interest for intraarterial treatments of the liver as perfusion based imaging techniques, such as volume perfusion CT (VPCT), dynamic contrast enhanced ultrasound, or dynamic contrast-enhanced MRI (DCE-MRI), are currently used for peri-interventional diagnostics [12–15].

First experiences with this technique in the evaluation of HCC have been published recently [16–18]. The applied algorithm in our study allows an acquisition of perfusion data using new flat detector generation with 16 bit for 4-fold better low contrast resolution, which is of great interest especially in parenchymal imaging (Artis Q, Forchheim, Siemens Healthcare). Compared to previous studies, the detector readout rate has become faster so that the acquisition time has been slightly shortened to approximately 2 s allowing improved patient comfort and avoiding potential motion artefacts.

The aim of this study was to compare PBV-based cross-sectional images acquired immediately before performing drug-eluting bead (DEB) TACE to pre-interventional conventional cross-sectional images (MRI, CT) performed for tumour staging. Furthermore, the feasibility and accuracy of PBV acquired images by C-arm CT using newly developed algorithms embedded in a new flat detector generation should be evaluated and investigated concerning its potential in the detection of tumour feeding vessels in comparison to conventional angiography.

## Methods

### Study population

A total of 29 patients suffering from early or intermediate stage HCC (median age, 73 years, range, 62–77 years) and undergoing TACE in our institution between January and November 2014 were included in this retrospective study. Indication for TACE was determined by our local gastrointestinal tumour board consisting of an experienced radiologist, a surgeon, a hepatologist and a pathologist. Patients with intermediate stage HCC (BCLC B) and early stage HCC (BCLC A) were included. TACE in BCLC A was performed as bridging therapy or as adjuvant therapy if curative treatment, such as radiofrequency ablation or surgery, was not possible. Exclusion criteria in this study were determined considering CIRSE guidelines [19] and included main portal vein thrombosis, bilirubin level above 2 mg/dl, creatinine level above 2 mg/dl, poor functional status (ECOG > 2), extrahepatic tumour spread, and limited liver function (Child-Pugh stage C, encephalopathy, ascites). All interventions were performed by the same board certified interventional radiologist. Underlying causes for the development of HCC were Hepatitis-C-virus infection (*n* = 9), Hepatitis-B-virus infection (*n* = 2), alcohol abuse (*n* = 9), or other causes (*n* = 5). In six patients, the underlying cause for developing HCC was cryptogenic. Detailed patient information is given in Table 1. Due to the retrospective nature of the study informed consent for retrospective data evaluation was waived by the local institutional review board (IRB).

**Table 1:** Baseline patient characteristics

Data		Value
Age (years)		
	Mean (SD)	69.83 (9.06)
Sex		
	Male	24
	Female	5
Child-Pugh_Classification		
	Child A	19
	Child B	10
Serum AFP level (ng/mL)		
MELD score		
	Mean (SD)	9.77 (3.06)
No. of nodules		
	1	11
	2	6
	3	8
	4	3
	> = 5	1
Lesion size (mm)	mRECIST	
	Mean (SD)	32.33 (21.24)
	Range	16.25 to 44.5
Lesion margins		
	Capsulated	33
	Infiltrative	31
Previous of TACES		
	Yes	15
	No	14
Epirubicin hydrochloride dose (mg)		
	Median	40
	Range	25 to 50
Interval between baseline CT and TACE (d)		
	Mean (SD)	15.72 (14.01)

### Pre-interventional imaging

All patients underwent cross-sectional imaging (MRI, *n* = 10, or CT, *n* = 19) before TACE (median, 16 days before TACE, range, 1–25 days), observing the national guidelines for the assessment of HCC [20]. Multiphase CT included non-enhanced, arterial (30 s after injection of contrast media) and portal venous phase (70 s after injection of contrast media) and was performed at a 128 row detector CT (Definition AS+, Siemens, Germany). In general, MRI included at least a T2 weighted turbo-spin-echo sequence as well as an unenhanced and three dynamic contrast enhanced T1 weighted gradient-echo sequences acquired at a field strength of 1.5 T (Avanto, Siemens, Germany). Patients received 0.1 ml gadobutrol per kilogram of body weight (Gadovist 1,0 mmol/ml, Bayer Healthcare, Germany). Arterial phase was acquired using bolus tracking.

All HCC lesions were classified concerning morphology in diffuse (*n* = 31) or encapsulated (*n* = 33) lesions. Encapsulated HCC is defined as a predominantly round lesion with presence of a capsule and remarkable washout in cross-sectional imaging. Diffuse HCC lesions are defined as predominantly irregular or lobular lesions without a capsule [21].

CT and MRI evaluation consisted of detection of number and extent of HCC lesions. All images were assessed concerning the modified Response Evaluation Criteria in Solid Tumours (mRECIST) for the presence and extent of HCC [22]. Maximum cross-sectional diameter of viable tumour mass was processed, preferably in an arterial phase. Images analysis concerning mRECIST was performed retrospectively and blinded to evaluation of angiographic and C-arm CT data sets by the board certified radiologist.

### Conventional angiography and chemoembolization

The arterial system was accessed through the common right femoral artery. After arterial puncture (19 G needle), a 4 F sheath (Terumo, Leuven, Belgium) and a 4 F straight catheter (Terumo, Leuven, Belgium) were introduced. An aortography was performed to assess the number and origin of hepatic arteries and for detection of abnormal anatomic blood supply of the liver in cases receiving first treatment, especially if anatomic blood supply was not clear from cross-sectional imaging. Furthermore, potential parasitic tumour supply was excluded or in case of presence of such a blood supply, these vessels were occluded by coils, immediately before performing chemoembolization. Afterwards, a 4 F Cobra (C2) or sidewinder (SIM1) configured catheter (Cordis, Bridgewater, New Jersey, USA) was introduced in the celiac trunk and coeliacography was performed. For selective catheterization of hepatic arteries, a 2,7 F coaxial microcatheter was used (Progreat, Terumo, Leuven, Belgium). Superselective chemoembolization was performed using epirubicin-loaded DC Bead particles (100–300 μm, Terumo, Leuven, Belgium). Loaded microspheres were injected slowly until near stasis was reached. After a time interval of approximately 10 min, a selective control angiography was performed. Number of HCC lesion and tumour feeding vessels were analysed in selective angiogram of the common hepatic artery (corresponding to catheter positioning of C-arm CT).

### C-arm CT PBV measurement and image processing

All interventions were performed using a robotic angiographic suite (Artis Zeego Q, VE 40 A, Siemens, Forchheim, Germany). For acquisition of PBV maps, an unenhanced rotation (mask run) and contrast enhanced rotations (return and fill run) were acquired (Acquisition time per rotation 4 s, total examination time 16 s, 90 KV, 200 ° total angle, 0.8° per frame, 248 frames, matrix 616x480 pixel, flat panel pixel size 616 μm, dose 0.36μGy per frame). For the contrast enhanced rotation, a total of 30 ml diluted contrast media [7,5 ml Ultravist 370 (Bayer Schering, Leverkusen, Germany) and 22,5 ml saline solution] was injected by an automated power injector (Accutron-HP-D, Medtron, Saarbrücken, Germany) with a flow rate 3 ml/s. Contrast injection was manually triggered after mask run, to guarantee a contrast enhanced acquisition in a steady state of liver perfusion, according to a previous study on PBV of the liver [16]. As blood volume refers to the amount of blood which is present at a given moment, it can be assumed to be constant during the time of acquisition. This makes the calculation of CBV from only two measurements possible: a base-line (mask) acquired before contrast administration and the contrast distribution (fill) after contrast injection. Acquired data allowed reconstruction of non-enhanced images, contrast enhanced images (arterial phase) including tumour feeding vessels, and PBV maps.

Acquired data were sent to a commercially available workstation (Syngo XWP, Siemens Healthcare) and reconstructed automatically. The software adopted the same post-processing workflow as previously described in the literature [23]. The mask and the fill run were reconstructed and subtracted. Non-rigid registration algorithm was performed to mitigate the motion between the two runs. The steady-state arterial input function value was calculated from an automated histogram analysis of the vessel tree. A final scaling was then applied to the dataset to account for the arterial input value. In the end, a smoothing filter was applied to the images to reduce pixel noise.

All processed PBV maps were analysed concerning number and extent of HCC lesions, whereas the number of tumour feeding vessels was investigated in PBV maps and arterial phase C-arm CT images. Retrospective evaluation of number and extent of HCC lesions were performed blinded to any results of pre-interventional cross-sectional imaging. Assessment of tumour feeding vessels were performed within the angiographic session in C-arm CT acquisition and conventional angiogram by a board certified interventional radiologist as all detected vessels were immediately verified by super selective angiography which means at least segmental angiograms according to CIRSE guidelines [19].

### Statistics

Statistics were performed using the statistical software Package JMP (SAS, Cary, NC). Median and interquartile ranges are given for descriptive statistics. The Wilcoxon signed rank test for non-normal distributed data was applied to assess the level of significance. A *p*-value lower than 0.05 was accepted as a significant difference. In case of categorical data Cohen’s κ was processed to evaluate agreement of different methods. In case of numerical data a Bland-Altman-plot was performed and mean difference (MD) as well as 95 % confidence intervals (95 % CI) were given. Correlation between different measurements and methods was calculated using Spearman’s correlation coefficient ρ.

## Results

### Lesion detection in PBV maps and pre-interventional imaging

**Fig. 1:**
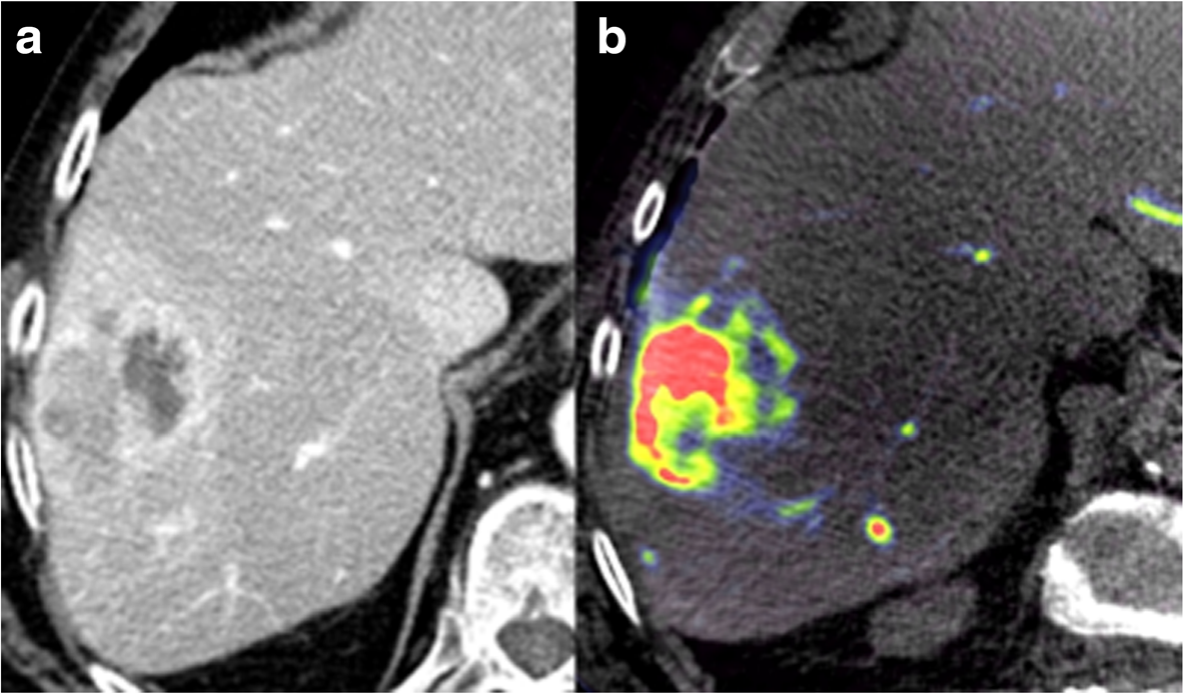
**a** Cross-sectional CT imaging (arterial phase) of an encapsulated HCC lesion with central necrosis. **b** Corresponding real-time PBV map gained during C-arm CT acquisition reveals an inhomogeneous hypervascularised HCC lesion in liver segment VII

**Fig. 2:**
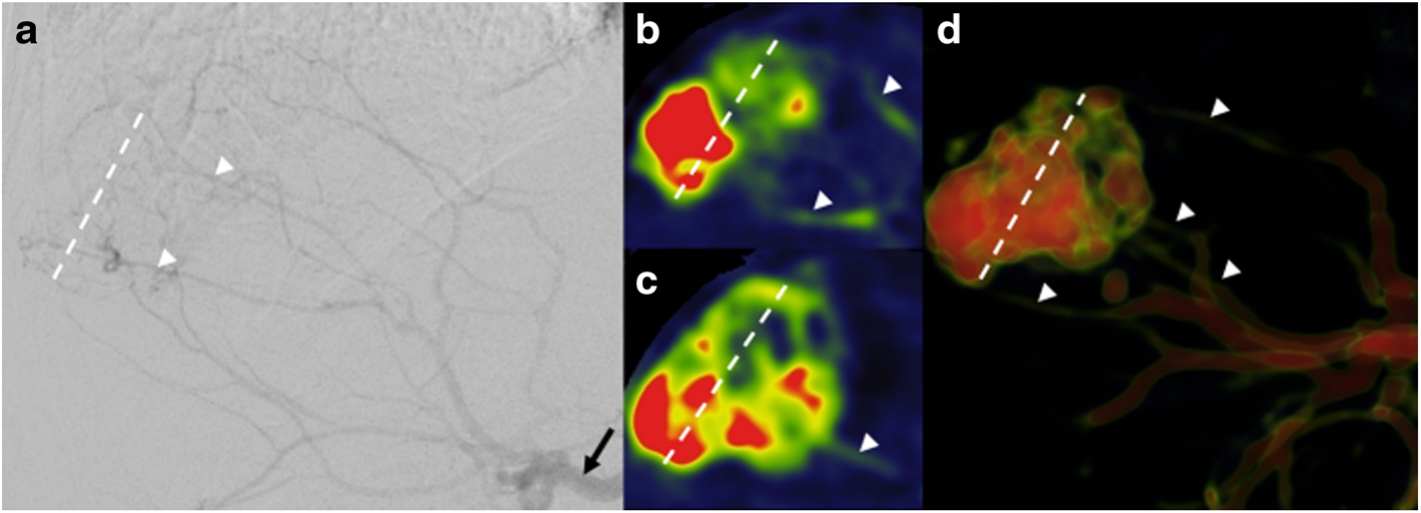
**a** Angiographic overview shows a hypervascularised HCC lesion in Segment VII (white line) with multiple tumour feeders (white arrows). Extent and location of tumour feeders is confusing. The black arrow marks the right hepatic artery. **b**–**d** 2D coronar (**b**, **c**) and 3D (**d**) PBV map reconstructions after C-arm CT acquisition facilitates analysis of location and extent of tumour feeding vessels

PBV-based C-arm CT was feasible in all patients and provided diagnostic quality in all cases. All HCC typical lesions (64/64, lesion diameter >10 mm) detected in pre-interventional imaging have been identified in PBV maps (κ = 1, *p* = 1.0) (Fig. 1). 49/64 lesions were detected in conventional angiography (κ = 0.77, p = 0.001). A total of 90 tumour feeding vessels were detected in PBV maps and arterial phase C-arm CT compared to 64 vessels in conventional angiography (κ = 0.71). This represents a median of 3 (range 2 to 4) tumour feeding vessels detected by PBV maps compared to a median of 2 (range 1 to 3) tumour feeding vessels seen in conventional angiography (*p* < 0.0001) (Fig. 2). All detected vessels were verified by super selective angiography. No additional tumour feeding vessels were seen in the super selective approach. Detailed information about number of tumour feeders and lesions in C-arm CT and corresponding angiogram are summed up in Table 2.

**Table 2:** Detection of HCC lesions and tumour feeding vessels

	Feeders		Lesions	
	Overall	Median (range)	Overall	Median (range)
Angiogram	64	2 (1–3)	49	1 (1–2.5)
C-arm CT/PBV maps	90	3 (2–4)	64	2 (1–3)
CSI	n.a.	n.a.	64	2 (1–3)

Detection of HCC lesions and tumour feeding vessels in C-arm CT/PBV maps and corresponding angiogram (common hepatic artery) as well as detection in pre-interventional cross-sectional imaging (CSI)

**Fig. 3:**
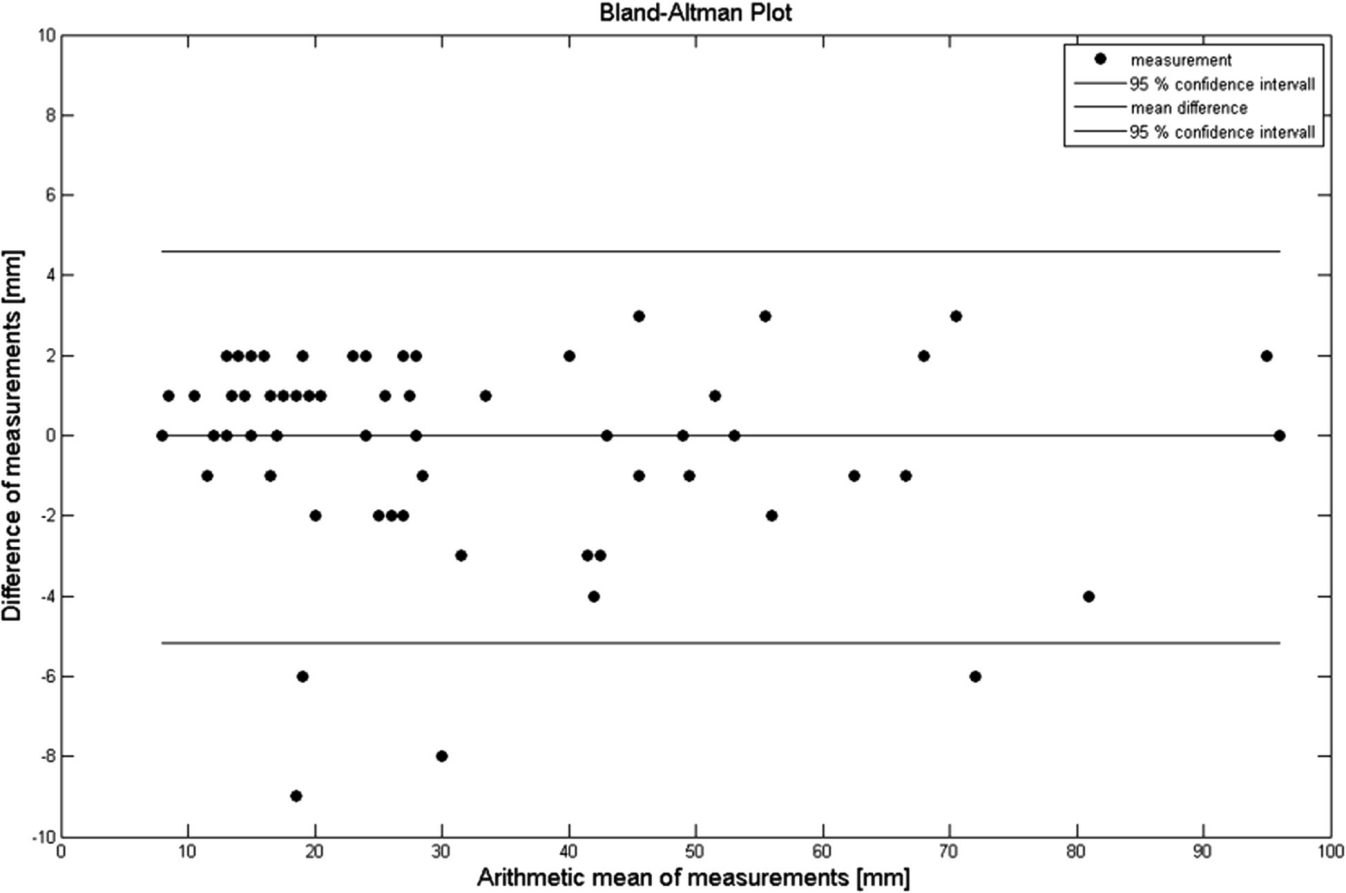
Bland-Altman-plot comparing maximum viable tumour diameter detected in pre-interventional cross-sectional imaging and PBV maps

Comparison of maximum cross-sectional diameter of all lesions in PBV maps and pre-interventional imaging revealed a maximum diameter of 32.61 (21.44) mm in PBV maps and 32.33 (21.24) mm in pre-interventional imaging (*p* = 0.88, MD = −0.28 mm, 95 % CI = −5.1 to 4.6 mm). Both method showed excellent correlation (rho = 0.98, *p* < 0.0001). The Bland-Altman plot is shown in Fig. 3.

### PBV in diffuse, encapsulated HCC lesions and after previous treatment

Overall, PBV was 18.25 (6.22) ml/100 ml in HCC lesions, compared to normal liver parenchyma with PBV of 2.89 (1.16) ml/100 ml (*p* < 0.0001, mean difference −15.36 ml/100 ml). HCC lesions having received a previous TACE session revealed no significant difference in PBV as compared to non-treated HCC lesions (p = 0.09), with a tendency towards higher PBV values in non-treated HCC lesions, revealing a mean PBV of 19.66 (6.88) ml/100 ml for non-treated lesions, compared to a mean PBV of 17.08 (5.44) ml/100 ml for pre-treated lesions. Concerning liver parenchyma, there was no significant difference in pre-treated and firstly treated parenchyma (*p* = 0.89). Comparison between diffuse and encapsulated HCC lesions revealed a significantly higher PBV in encapsulated lesions, 21.22 (6.29) ml/100 ml, compared to diffuse lesions with a mean PBV of 15.09 (4.35) ml/100 ml (*p* = 0.0001). Normal liver tissue showed no significant difference in PBV between the groups, with a mean PBV for encapsulated HCC group of 3.05 (1.22) ml/100 ml and a mean PBV for diffuse HCC group of 2.71 (1.09) ml/100 ml (*p* = 0.23).

### PBV and maximum diameter

Comparison of maximum diameter assessed in pre-interventional imaging and PBV values revealed no correlation regarding all HCC lesions (rho = 0.23). Separate analysis of diffuse and encapsulated HCC lesions also showed no correlation between maximum diameter and PBV for encapsulated HCC lesions (rho = 0.18). However, in diffuse HCC lesions, a moderate correlation between PBV values and maximum diameters was seen (rho = 0.52).

## Discussion

This retrospective study investigates the applicability and the clinical potential and value of PBV maps as well as non-enhanced and contrast enhanced C-arm CT acquisition in pre-treatment evaluation of TACE in HCC.

Evaluation of the amount and location of HCC lesions and tumour feeding vessels is of great importance in treatment planning of TACE. Conventional angiography allows only a two dimensional approach to tumour volume and feeding vessels, whereas most pre-interventional assessments are based on three-dimensional data, e.g., MRI and CT acquisitions. C-arm CT provides an exciting technology for interventional radiology, as it allows 3D real-time imaging [24].

Virmani et al. investigated the usefulness of C-arm CT to optimize the catheter positioning during conventional TACE. Based on C-arm CT assessment, a correction of catheter positioning was necessary in 7 of 18 patients [25]. Kakeda et al. showed that C-arm CT added useful information for catheter positioning in 81 % of the cases [7]. These results are supported by our study which showed an advantage in the detection of tumour feeding vessels for C-arm CT PBV measurement and arterial phase imaging in comparison to conventional overview angiography. Furthermore, anatomic location and extent of smaller or less vascularised HCC lesions was more easily appreciated using C-arm CT PBV measurement when compared to conventional overview angiography. Fifteen supplementary lesions (23 %) were detected using PBV maps. All supplementary lesions were confirmed in pre-interventional cross-sectional imaging. These results are comparable to a previous study concerning C-arm CT which reported a detection rate of supplementary lesions of 15 % [8]. In summary, use of C-arm CT PBV measurement including non-enhanced and contrast enhanced anatomical images results in a more appropriate tumour targeting during DEB-TACE.

In addition to anatomic information in terms of tumour extent and location, C-arm CT PBV measurement provides quantitative real time information about tumour perfusion and vascularisation.

Previous studies reported on treatment effects of conventional TACE on blood volume (BV) assessed by VPCT. Lesions reported to have a partial response showed decreased BV of about 50 % compared to pre-treatment values [15, 26]. In our study, pre-treated HCC lesions tended to have lower PBV compared to HCC lesions which received TACE for the first time. The reduction of PBV was about 15 %. The difference is probably based on two facts. Firstly, we have only cross-sectional data at one time point. The difference might be more obvious in longitudinal data sets in which pre- and post-treatment PBV values of the same subject would be available. Secondly, the time interval between baseline and follow-up was only four weeks in the reported studies [15, 26]. The normal time interval between consecutive TACE sessions in our study ranges between 6 and 12 weeks. C-arm CT with additional PBV maps has only be evaluated in one previous study which focused on pre-treatment PBV evaluation in comparison to VPCT [16]. C-arm CT PBV measurement was described as a feasible method which allows to process hepatic blood volume comparable to VPCT data. Our study additionally assessed tumour size which has also be reported to be a relevant parameter in outcome of patients suffering from HCC [27]. C-arm CT PBV measurement provides a real time assessment of tumour size comparable to pre-interventional cross-sectional imaging and seems therefore to be appropriate for assessment of tumour extent applying mRECIST criteria. The mean PBV value for HCC lesions and liver parenchyma observed in our study was 18.25 ml/100 ml vs. 2.89 ml/100 ml, respectively. This was slightly lower compared to previous study (25.9 ml/100 ml vs. 4.2 ml/100 ml). The higher PBV of liver parenchyma might be due to the fact that in the earlier study, mainly Hepatitis-B associated tumours were included and it is well known that different underlying diseases provoke a different grade of liver fibrosis [28]. The difference in PBV concerning HCC lesions could be associated with different tumour morphology (number of diffuse/encapsulated HCC). Furthermore, the previous study did not include pre-treated HCC lesions as we did in our study [16]. Both pre-treatment situations and diffuse HCC seem to be associated with lower PBV values. Whereas the lower PBV in pre-treated lesion might underline a therapeutic effect, the lower PBV values as well as the association between maximum lesion diameter and PBV values in diffuse lesions could be related to the different microvessel structure in expansive growing HCC [29].

In summary, anatomic as well as quantitative information gained in PBV maps and non-enhanced as well as contrast enhanced C-arm CT acquisition could be used for a further optimization of chemoembolic treatment of HCC. Miyayama et al. already showed the usefulness of tumour feeder detection by C-arm CT for an ultraselective approach in TACE [30].

The main limitation of this study is its retrospective design. Only a small sample size of 29 patients is investigated in a cross-sectional approach. Further prospective longitudinal trials have to be performed to analyse potentials of PBV in prediction of outcome and survival. Another limitation is the use of two different imaging techniques in pre-interventional assessment. This limitation is due to the retrospective design of the study and its acquisition in clinical routine. The aim of this study was to underline that PBV maps generated from unenhanced and contrast enhanced C-arm CT data provides a useful technique in real-time assessment of HCC. A histological correlation was not possible, as this was a human in-vivo study.

## Conclusion

C-arm CT PBV measurement provides an appropriate tool for pre-interventional real time assessment of the location and extent of HCC tumours with a diagnostic accuracy comparable to pre-interventional conventional cross-sectional imaging. Compared to simple contrast enhanced C-arm CT, PBV maps additionally enable a quantitative assessment of hepatic and tumour blood volume, which could be of further interest in a real-time treatment assessment and planning in DEB TACE of HCC. Furthermore, C-arm CT acquisition in combination with PBV measurement seems to be superior in localisation of tumour anatomy and feeding vessels when compared to conventional overview angiography.
